# The Importance of Coordinated Actions in Preventing the Spread of Yellow Fever to Human Populations: The Experience from the 2016-2017 Yellow Fever Outbreak in the Northeastern Region of São Paulo State

**DOI:** 10.1155/2019/9464768

**Published:** 2019-05-19

**Authors:** Márcio Junio Lima Siconelli, Danillo Lucas Alves Espósito, Nathália Cristina Moraes, Julia Maria Ribeiro, Lívia Perles, Maria Angélica Dias, Adolorata Aparecida Bianco Carvalho, Karin Werther, Natália Coelho Couto de Azevedo Fernandes, Silvia D'Andretta Iglezias, Karina Paes Bürger, Benedito Antonio Lopes da Fonseca

**Affiliations:** ^1^Department of Internal Medicine, Ribeirão Preto Medical School, University of São Paulo FMRP/USP, Av. Bandeirantes, 3900, Vila Monte Alegre, Ribeirão Preto, 14049-900 São Paulo, Brazil; ^2^Veterinary Preventive Medicine and Animal Reproduction Department, Faculty of Agricultural and Veterinary Sciences, Universidade Estadual Paulista “Júlio de Mesquita Filho” (FCAV/Unesp), Access Way Prof. Paulo Donato Castellane s/n, Jaboticabal, 14884-900 São Paulo, Brazil; ^3^Animal Pathology Department, Faculty of Agricultural and Veterinary Sciences, Universidade Estadual Paulista “Júlio de Mesquita Filho” (FCAV/Unesp), Access Way Prof. Paulo Donato Castellane s/n, Jaboticabal, 14884-900 São Paulo, Brazil; ^4^Municipal Health Secretary, Av. General Glicério, 569, Center, Jaboticabal, 14870-520 São Paulo, Brazil; ^5^Nucleus of Pathological Anatomy, Pathology Center, Adolfo Lutz Institute of São Paulo, State Secretary of Health, Av. Dr. Arnaldo, 351, 7° Floor, 01246-902 São Paulo, Brazil

## Abstract

Yellow fever (YF) is a zoonotic arthropod-borne disease that is caused by the yellow fever virus (YFV) and characterized by a sylvatic and urban cycle. Its most severe presentation is manifested as a hemorrhagic disease, and it has been responsible for thousands of deaths in the last decades. This study describes the public health approaches taken to control the 2016-2017 YF outbreak in nonhuman primates (NHPs) that took place in the northeastern region of São Paulo state, Brazil. NHPs recovered from the field were necropsied, and YF diagnoses were made at the Laboratory of Molecular Virology, Ribeirão Preto Medical School and the Center of Pathology, Adolfo Lutz Institute of São Paulo. NHP samples were inoculated into Vero cells for YFV isolation. RNA extraction was performed directly from NHP tissues and tested by RT-qPCR. YFV-positive samples were confirmed by sequencing. Based on the rapid RT-qPCR results, surveillance actions were implemented in the entire region. Confirmatory histopathology and immunohistochemistry for YFV were also performed. Among nine NHPs, gross hepatic involvement was observed in six animals, five of which were YFV-RT-qPCR-positive. One YFV was isolated from the serum of an infant NHP. YFV RNA sequences diverged from the virus responsible for the last epizootic that occurred in São Paulo state, but it was similar to the current Brazilian epizootic. Public health actions included dissemination of information on YF transmission, investigation of the probable location of NHP infection, characterization of the environment, and subsequent creation of the blueprint from which prevention and control measures were implemented. The YFV sylvatic cycle occurred in the periurban areas of the northeastern region of São Paulo state, but no human cases were reported during this period, showing that integrated actions between human, animal, and environmental health professionals were critical to restrain the virus to the sylvatic cycle.

## 1. Introduction

It is estimated that 60% of disease-causing pathogens in humans are zoonotic, and most of them are maintained in the wild through enzootic cycles [1, 2]. One example of these pathogens is the yellow fever virus (YFV), which causes yellow fever (YF), an acute and noncontagious infectious disease that affects animals and humans. YF is characterized by fever, jaundice, and hemorrhage, and it is responsible for the death of hundreds of people. YF remains enzootic in the tropical forests of America and Africa causing epizootics with known periodicity or epidemics of greater or lesser impact on public health [3–6]. Its reemergence in the areas outside of the Amazon region is concerning for public health authorities in the Americas and throughout the world.

In the Americas, transmission occurs through the bite of infected female hematophagous mosquitoes from the *Culicidae* family, especially by the *Aedes aegypti* species, in the urban area, and *Haemagogus* and *Sabethes* genera in the wild environment, characterizing two cycles of transmission: an urban and a sylvatic cycle [6–9]. However, the existence of a possible new vector in Brazil, *Aedes albopictus*, has been suggested as susceptible to infection by YFV [10–14].

The sylvatic cycle of YF transmission involves nonhuman primates (NHP) and sylvatic mosquitoes [15, 16]. The NHP species that are present in South America are mostly susceptible to YFV infection [6]. Among neotropical monkeys, the disease occurs in an epizootic manner, especially with the genus *Alouatta*, in which a high mortality rate is observed, and this represents a concern regarding species conservation and ecological balance in the area affected by YFV [6, 17–21]. Many other primates are severely affected, such as those of the genera *Ateles*, *Aotus*, *Saguinus*, *Cebus*, *Sapajus*, *Callithrix*, and *Callicebus*. Although monkeys of the genera *Cebus* and *Sapajus* are easily infected, they usually have a subclinical infection with low lethality, and they generally develop protective immunity, as shown by the presence of neutralizing antibodies, which indicates probable infection and survival [6, 22, 23].

The YFV is the prototype of the *Flavivirus* genus from the *Flaviviridae* family, which has genetic material that consists of a positive-sense, single stranded RNA [24]. Its genome encodes three structural (C, *pr*M, and E) and seven nonstructural (NS1, NS2A, NS2B, NS3, NS4A, NS4B, and NS5) proteins [25, 26]. Although only one serotype of the YFV has been described, there are seven genotypes that carry small genetic changes, two from the Americas (South America 1 and South America 2) and five from Africa (West Africa 1, West Africa 2, East Africa 1, East Africa 2, and Angola), but no difference in the disease presentation is observed among them [27–29].

In Brazil, the most frequent genotype is South America 1 (SA1), although five other lineages that derived from SA1 has been identified (SA1A to SA1E), with SA1E previously recorded in the 2008 epidemic in São Paulo state [9, 24, 30–32]. Recently, a YFV genome from a virus that was isolated in Espirito Santo state during the 2017 outbreak was sequenced in its entirety, and changes were found to eight amino acids that were located in the C, NS3, and NS5 proteins [32].

The last case of urban YF in Brazil was reported in 1942 in the Amazon region, and since then, no records of urban transmission have been confirmed [8, 24]. However, the risk of YF reurbanization exists because *Ae. aegypti* is widely distributed in the country.

In the recent history of São Paulo state, three major sylvatic outbreaks have been recorded. In 2008, two human deaths, from autochthonous cases, were reported in the northeastern region of the state [33–35], and in 2016, Brazil reported six confirmed human cases of YF to the World Health Organization (WHO), with two deaths occurring in São Paulo state [36–38]. According to the São Paulo State Health Department, 227 animals with suspected YFV infection were reported in the state up to December 2016, and among those, 24 were positive for YFV in the northern region of the state [36]. In 2017, 21 autochthonous human cases were confirmed up to June, and nine patients died. During this period, 110 of 643 NHP were also infected with YFV [36].

In the last two years, Brazil has experienced the largest outbreak of YF in the last 70 years, a situation that has alarmed the population and health authorities mainly because of the virus dissemination, which reached areas that were previously classified as free of YFV circulation [21, 32, 39, 40].

Because of the absence of records on YFV infections in NHP since 1935 [39] in the northeast-central region of São Paulo state, the objective of this manuscript is to report the features of the recent YF outbreak in NHPs in this region of Brazil, the molecular characterization of a YFV isolated during this period, and the importance of these findings to initiate coordinated measures among all public health services to prevent the occurrence of human cases.

## 2. Materials and Methods

### 2.1. Description of the Study Site

**Figure 1:**
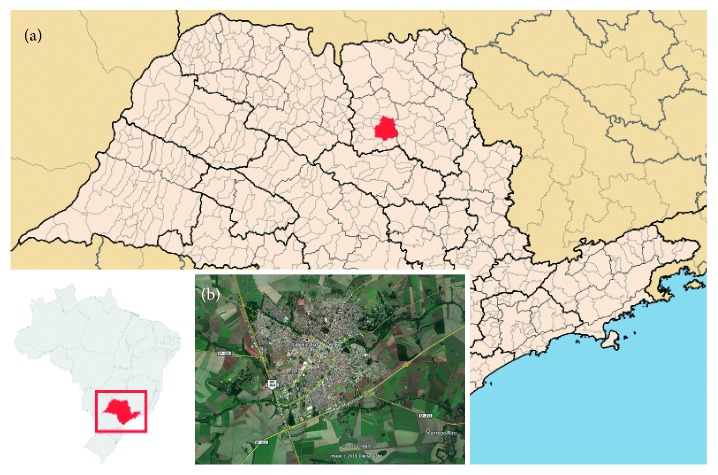
(a) Map from the study site. In the smaller image, São Paulo state is in red inside Brazil's map and the larger image is the São Paulo state map, including the study site (Jaboticabal/SP) that is highlighted in red. (b) Aerial satellite view of the urban and rural areas of the municipality of Jaboticabal/SP. Sources: (a) http://www.skyscrapercity.com/showthread.php?t=1513060; (b) Google Earth, 2017.

The study site described here is located in the northeastern region of São Paulo state, at the coordinates 21°15′18.00″ south and 48°19′19.20″ west (Figure 1). The area is characterized by long green corridors, extending from rural to the urban areas. The vegetation in the region is characterized by the Brazilian cerrado biome (regarded as the Brazilian Savanna), which is mostly residual ciliary forest including areas of permanent preservation. The intense exploitation of sugarcane cultures left fragments (“islands”) of forested areas and in some parts without direct communication between them, forming true mosaics of vegetation. The average annual rainfall is approximately 1423.7 mm, accumulating in the months of January, February, March, October, November, and December. In addition to the high precipitation, the average temperature is also high, reaching an average of 29.1°C. The month of October stands out with the highest temperatures, with an average of 30.9°C [41].

### 2.2. Necropsy and Collection of Biological Material

Necropsy and collection of biological samples were performed at the Service of Pathology of Wild Animals, School of Veterinary Medicine, São Paulo State University (FCAV/Unesp), following an established protocol for necroscopic examination of NHPs [42, 43]. Most of the necroscopic examinations were performed as soon as the NHP arrived at the Pathology Service to avoid organ degradation. However, some animals were either refrigerated (between 2°C and 8°C) or frozen (−20°C) before the necroscopic examination.

Concomitantly, biological samples were collected according to the “Guide for the Surveillance of Epizootics in Nonhuman Primates and Entomology Applied to Yellow Fever” [42, 43] and subsequently stored. The samples were sent to the Laboratory of Molecular Virology, Ribeirão Preto Medical School, University of São Paulo, Ribeirão Preto, São Paulo, for virologic and molecular testing and to the Center of Pathology of Adolfo Lutz Institute of São Paulo for histopathological and immunohistochemical testing. These test results were required to make a diagnosis of YF.

Tissue fragments of approximately 0.5 cm by 2.0 cm were collected and divided in two sections. One section was placed in 10% buffered formaldehyde solution and kept at room temperature for subsequent encasement in paraffin blocks and histopathological testing. The other section of the sample was frozen at −20°C and subsequently stored at −70°C. The main organs sampled were the liver, spleen, lung, brain, heart, and kidneys. Based on the viability of the carcass, other tissues were harvested, such as the stomach, lymph nodes, adrenal glands, intestines, blood, bladder, and gonads.

### 2.3. Histopathological and Immunohistochemical Diagnosis

Slides that were 3 *μ*m thick were made from formalin-fixed and paraffin-embedded tissues and were stained with hematoxylin and eosin (H&E) before microscopic examination. The main lesions were described and classified according to intensity and distribution. Additional slides were deparaffinized, followed by antigen retrieval under pressure and incubation with primary anti-FA polyclonal antibody (produced in the Arthropod-borne Virus Laboratory at the Adolfo Lutz institute). Signal amplification was achieved using a polymer conjugated with an enzyme (MACH4 universal AP Polymer and Polymer MACH4™ universal AP, Biocare, Pike Lane, Concord, CA, USA) that was visualized using chromogen (Warp red™, Biocare, Pike Lane, Concord, CA, USA).

### 2.4. Molecular and Virologic Diagnosis

To optimize virus recovery, liver samples were chosen to be tested first because of the YFV tropism to the liver. RNA was extracted using the phenol-chloroform technique, and it was subjected to YFV-specific RT-qPCR. If the result was positive, other harvested tissues were subjected to RT-qPCR (TaqMan® Fast Virus 1-Step Master Mix, Applied Biosystems, Woodward St., Austin, TX, USA) to verify the tissues in which the virus was located. Primers used in both experiments were specifically designed for this study and are shown in Table 1. Samples with a positive result were subjected to Sanger sequencing (BigDye™, Applied Biosystems, Woodward St., Austin, TX, USA).

### 2.5. Surveillance

When the first cases were confirmed in October 2016, the Municipal Health Department was notified, and prevention and control strategies were implemented by public health officials. Strategic actions were based on selective house-to-house immunization throughout the rural areas in the region, mainly in the areas neighboring the location of the YFV-positive cases, to verify the population's vaccination status, control mosquito breeding sites in all constructed areas where animals were found, intensify surveillance for NHP death, implement georeferencing in the affected area, and conduct health education for local health professionals and the population. The coordinates of the NHPs were georeferenced with the help of the native GPS application iOS 10 (Apple, Cupertino, CA, USA). The data were then entered into the Google Earth 7.1.7.2602 software. All occurrences received at the local surveillance unit were recorded, including live or dead animals and carcasses.

### 2.6. Ethics

This study was approved by the FCAV/Unesp Ethics Committee on the Use of Animals (process no. 010001/17). The reported cases followed the guidelines for research, diagnosis, prevention, and control measures, according to the “Guide to Surveillance of Epizootics in Nonhuman Primates and Entomology Applied to Yellow Fever” [42, 43] and the “Health Surveillance Guide” [42]. Additionally, all the cases investigated were entered into the Notification of Injury Information System (SINAN) from the Ministry of Health of Brazil.

## 3. Results

### 3.1. Cases

In late October 2016, an infant male howler monkey (*Alouatta caraya*) (monkey #1) was found separated from his group in a country club located on the outskirts of the main city and within the study site, and it was sent to the Veterinary Medical Service of Wild Animals of FCAV/Unesp. After a thorough physical examination, it looked healthy, and it was kept under quarantine. Three days later, the monkey developed apathy, anorexia, weight loss, pale mucosae, and loss of consciousness. In the following days, there was an intense deterioration of its clinical status, and the veterinarians chose to perform euthanasia. During this period, the Environmental Military Police reported that another young male howler monkey, from the same region drowned in the lake at the same country club (monkey #2). It was removed from the water and taken to the FCAV/Unesp but subsequently died.

**Figure 2:**
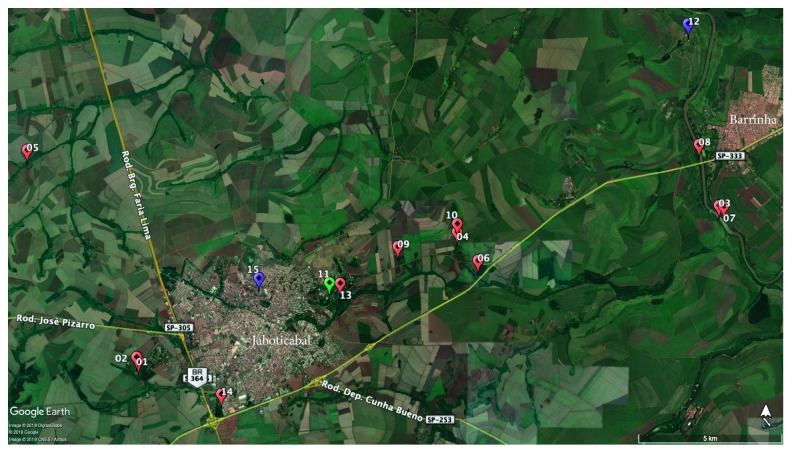
Satellite image of the study site with georeferenced notifications, between October 2016 and April 2017. Numbers 01 to 15 correspond to the sequence of notifications received during the period. Red dots represent animals that recently died, carcasses, or bones (*Alouatta caraya* species). The green dot represents an animal that recently died (*Sapajus nigritus* species). A purple dot represents a carcass of the *Callithrix penicillatta* (12) and *C. jacchus* (15) species. Source: Google Earth, 2019.

After these cases, other reports were received, and nine animals were sent to FCAV/Unesp. All animals were necropsied, including five howler monkeys (monkeys #3 to #7), one capuchin monkey (*Sapajus nigritus*; monkey #8), and a white-tufted marmoset (*Callithrix jacchus*; monkey #9). Monkey #9 had been kept as a pet by authorized owners (Table 2). The reporting areas are presented in the map below (Figure 2).

The first positive YF cases (monkeys #1 and #2) were found in the western part of the study site. All other animals were found in the eastern region (monkeys #3 to #8), including those from which we could not collect any samples because of the advanced stage of decomposition. Although monkey #3 had no evidence of YFV infection, it was considered positive for YFV infection based on epidemiologic criteria. In addition to the monkey deaths, area residents reported that NHP deaths had probably been happening throughout August and September.

Other reports were made in addition to the reported cases, for a total of nine animals, comprising eight howler monkeys and a black-tufted marmoset (*Callithrix penicillata*). It was not possible to perform a necropsy on these animals because only bones or carcasses that were in an advanced stage of decomposition were found. These animals were included in this study because they were found near the sites where the other monkeys that were infected with YFV were found. Additionally, previous ongoing animal surveillance had shown that it was unusual to find so many dead animals in such small area and in a short time period.

### 3.2. Necropsy Findings and Histopathological and Immunohistochemical Diagnosis

**Figure 3:**
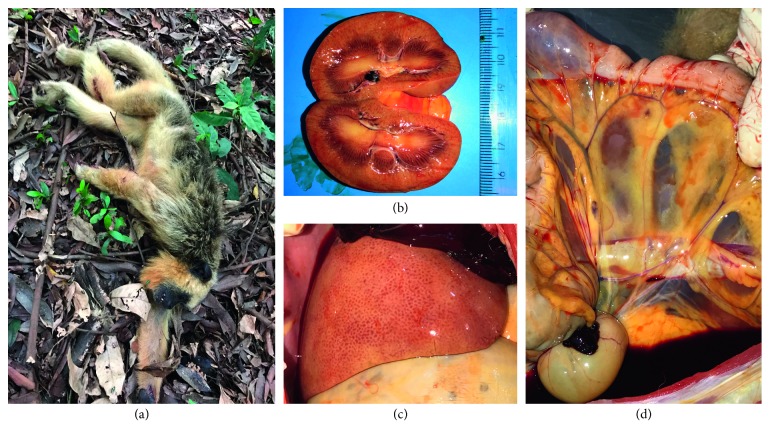
A female of *Alouatta caraya* species (monkey #4). (a) Record of the place where the animal was found. (b) Kidney midsagittal section, showing yellowish coloration. (c) Liver with yellowish coloration and reticular pattern evidenced. (d) Mesentery with yellowish coloration and intense hemorrhage.

Of the nine necropsied animals, five of them, all *Alouatta caraya*, had alterations that were suggestive of an acute infectious disease. External examination detected animals with a body weight within normal limits for the species and mucosa with intense yellowish color (ocular, oral, anal, and vaginal/preputial). An internal examination showed that the main organs affected were the liver, which was a normal size and had an evident reticular pattern with the color varying from pale yellow to intense yellowish; the kidneys were uniformly yellowish, in both the cortical and medullar regions, and without any change in size; and in some of these animals, the color of the omentum varied from yellow to intense orange. Two of the animals had hemorrhagic fluid and coagulated blood in the abdominal cavity (Figure 3).

Other findings, such as petechiae on the cardiac musculature, cerebral hemorrhage, splenomegaly, and yellowish staining on the walls of the gastrointestinal tract were also found. No similar gross pathological features were found in the livers of the other four necropsied animals, with the exception of a white-tufted marmoset that had yellowish regions on its liver.

**Figure 4:**
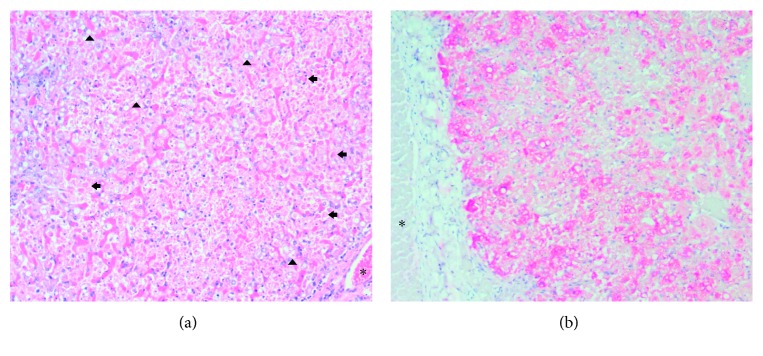
Histopathologic and immunohistochemical findings in the liver of a neotropical nonhuman primate, *Alouatta caraya* species (monkey #5), that died of YF in Jaboticabal, São Paulo, Brazil, 2016. Asterisks (^*∗*^) indicate the portal vein. (a) Moderate (diffuse) lytic necrosis with some Councilman-Rocha Lima (apoptotic) bodies (arrows), mild macrovacuolar steatosis (arrow head). Original magnification ×100, H&E staining. (b) Positive granular and massive intracytoplasmic immunolabeling for the yellow fever virus antigen, with panlobular distribution. Original magnification ×100; anti-YF virus (NDTV/IAL), Warp red™, counter staining with Harrys hematoxylin.

All five confirmed cases showed intense panlobular necrosis with acidophilic bodies (Councilman-Rocha Lima bodies), mild to moderate fat degeneration, and mild to moderate inflammatory infiltrate, mainly in portal tract, that rarely had neutrophils associated (Figure 4). Multifocal hemorrhage was present in one of the cases. Most of them presented lymphoid necrosis in the white pulp of the spleen and proteinosis in the renal tubules. The brain, lungs, and heart did not present remarkable findings. On immunohistochemistry, all cases showed a strong red immunolabeling that was limited to the hepatocyte cytoplasm, indicating a severe infection.

### 3.3. Molecular Diagnosis and Epidemiology

Eight samples were tested at the LMV-FMRP/USP (Laboratory of Molecular Virology, Ribeirão Preto Medical School at the University of São Paulo). Hepatic tissue from all NHPs was tested, except for monkey #1, from which it was only possible to collect and extract RNA from serum. RT-qPCR detected YFV in five samples (monkeys #1, #2, #4, #5, and #9), confirming infection by YFV. The other samples were negative (Table 2). The samples from monkey #2 were tested at the Adolfo Lutz Institute of São Paulo, and YFV infection was also confirmed. Several other tissues for monkeys #4, #5, and #9 were also RT-qPCR-positive for YFV. A summary of YFV detection in these samples is presented in Table 3.

After a positive RT-qPCR result, 1,000-bp fragments including the capsid, prM, and E protein regions of the genome were sequenced. Sequences were submitted to the GenBank database (accession numbers: MF443184, strain JabSPM01; MF443185, strain JabSPM02; MF443186, strain JabSPM03; and MF443187, strain JabSPM04). A YFV from one of the monkey samples (4283912, strain JabSPM02) was isolated in Vero cells.

### 3.4. Surveillance

Based on the positive laboratory results, strategies were implemented to avoid the virus spreading into the population in the adjacent rural areas and especially into the urban area.

An intensive house-to-house vaccination program was conducted in the rural area of the epizootic region, initially targeting the locale where the first positive cases were found. During vaccination, health education was also provided for the area residents using a personal approach, and information leaflets about the disease were handed out. Control measures were then extended into neighboring areas.

To evaluate the vaccination status of the population and, if necessary, to perform mass vaccination, state and city governments established a vaccination “*D*” day, which was about 10 days after beginning of the house-to-house vaccination. On that day, approximately 3,000 doses of the vaccine were administered to the population. Combining the vaccination campaign that started about 2 weeks before the “*D*” day and the vaccinations performed on “*D*” day, more than 10,000 doses of YF vaccine were used to immunize people living in high-risk areas, either for the first time or to boost the immune response against YF. House-to-house vaccination in the rural area of the epizootic location lasted for 33 days and was completed in early 2017.

In addition to these actions, surveillance of NHP health status within the study site was intensified. Monkey groups living in small forested areas in urban and periurban environments were tracked and monitored, looking for the presence of either diseased or dead animals during the epizootic period.

## 4. Discussion

Since the 1990s, the northeastern portion of São Paulo state has been considered a high-risk area for YF transmission, and vaccination of people in this area has been recommended since that time [17]. In Brazil, a high-risk area is defined as a location where the YFV circulates either continuously or intermittently. After approximately 7 years without YF cases in São Paulo state [31], the first human death was reported in March 2016, in a wooden area located about 100 miles from the area described in this study. After that case in July 2016, *C. penicillata* was found in a city near our study site, which was later confirmed as YFV positive. In October of the same year, an epizootic broke out within the site described here, and samples were collected from nine of 18 NHP found, and YFV infections were confirmed in five of them. Samples were not collected from 9 NHP because of the advanced stage of decomposition. However, there was no other evidence for their cause of death besides inhabiting the area where the YFV-infected monkeys were found. During that time, the study site was one of the areas that had the highest number of reported YF cases in *A. caraya* (13/15; 86.7%), which was similar to numbers reported in the 2009 outbreak [6, 17, 34]. This is directly related to the susceptibility of this species, which is considered to be the main sentinel for the disease [6]. All NHP cases, except for the white-tufted marmoset, were located in periurban or rural areas.

The study site described here was characterized as small areas of remaining forest that had been either destroyed or preserved with changes in the native vegetation. Additionally, field investigations demonstrated that all animals were found near the riverbanks or streams. The first two YF-positive cases were found in the western part of the study site in October, and soon thereafter, other animal deaths were reported in the eastern region. During epidemiologic investigations, several NHPs' bones were found, mainly in the eastern region. However, it is not possible to establish a direct connection between the two regions, which are separated from each other by approximately 5 to 6 miles, a space that is occupied by a city of about 75,000 inhabitants, and there had been no record of free-living NHPs in that urban area. Additionally, people living in the rural areas reported that NHP deaths had been happening since August or September. Although some animals were found between late November and early December 2016, it is possible that the virus had been circulating already for months. The detection of a YFV-infected howler monkey in January 2017 (monkey #9), which was found dead between September and November 2016 but kept frozen for teaching purposes, reinforces this assumption.

Although improbable, human activity may have contributed to the spread of the virus to several areas, suggesting that this is of central importance in epizootic/epidemic outbreaks and it can act directly as a source of infection. This hypothesis is based on the fact that a transiently infected person in their viremic or incubation period can introduce the virus to areas with high vector population densities and increase the availability of vertebrate hosts, such as NHPs [6, 31, 39]. However, there has been no evidence for human infections in the study area, which is an observation that goes against the possibility of humans taking a central role in YFV transmission.

In Brazil, most of the municipalities have an infestation by the *Ae. aegypti*, *Ae. albopictus*, or both and show high levels of infestation, with R0 (basic reproduction number) >1, which indicates an initial phase of a dengue epidemic. The R0 for urban YFV transmission is 43% lower than that of the initial phase of dengue [44, 45]. To lower the risk of transmission, all mosquito control measures must be improved in urban areas, where people and the health authorities can act directly against the vector (i.e., drain accumulated water from containers and eliminate all mosquito breeding sites). In the sylvatic cycle, it is not possible to introduce any control measures for the mosquitoes because there is no way to remove all breeding sites from the trees due to their location in the canopy and that all mosquito species that can transmit the YFV have different habitats. Additionally, it is not acceptable to control these mosquitoes using insecticides. Because controlling the sylvatic mosquito population is difficult, and YFV vaccination is the only strategy for effective prevention that is available for people living in the rural or sylvatic areas.

Indirectly, destroying forested areas to construct human dwellings resulted in a stronger interaction between humans and the sylvatic environment (wild animals and many vector species). Because of this, NHPs and other animals started to move out of their natural habitat in search for food, water, and shelter, creating the opportunity for translocation of various pathogens that can potentially cause zoonotic diseases, such as YFV, between NHPs and humans [31, 46]. Although reurbanization of the disease has been speculated, because urban yellow fever is transmitted by *Ae. aegypti*, a mosquito that is highly prevalent in Brazil, YF reurbanization has not happened. However, many other hypotheses may try to explain the lack of reurbanization, such as genetic differences from the 1942 *Ae. aegypti* population, when it was eliminated, and the current *Ae. aegypti* population. Mutations in the YFV genome that occurred throughout the years may have changed the transmission patterns through acquisition of several new hosts in the sylvatic cycle. Furthermore, it is important to investigate the presence of the virus in populations of *Ae. aegypti* during seasonal period [14].

Other explanations besides the above hypothesis have been suggested for the emergence of apparently unprecedented NHP cases, such as maintenance of the virus in animal species other than NHP so that transmission is kept at low levels, and after 5 to 8 years of low activity, the virus can replicate in epizootic proportions [6, 47, 48]. The absence of YFV circulation during this period is due to the time that the monkey populations take to recover, and new susceptible animals are born. Additionally, YF-infected mosquito eggs remain viable in the environment by resisting harsh weather conditions and hatching under suitable climate conditions, which may contribute to the permanence of the virus in the wild [24, 39, 49]. Finally, illegal trafficking of wild animals can move the virus out of its sylvatic cycle and introduce it into virus-free areas [21, 42].

Thus, to control the sylvatic cycle of YFV and prevent dissemination from the area with confirmed cases, working perimeters of the investigation were defined from the places of probable infection. For places where laboratory-confirmed YF cases were found and those with the presence of either rivers or streams, there was a recommendation that house-to-house vaccination should take place within a 30-kilometer radius from the reported case. However, because of limited operational capacity of healthcare personnel, a radius of 15 kilometers was used, including the study site. This intervention was based on the observations made during previous outbreaks, such as the epizootic of Minas Gerais (2002/2003), which included the banks of the Rio Doce, and the epizootics in São Paulo, which occurred from 2000 to 2010 and involved other rivers (Rio Grande, Rio Cubatão, Rio Mogi-Guaçu, and Rio Paranapanema) [33]. The presence of forested areas bordering on rivers that often pass through urban areas may constitute a relevant risk factor for the disease. Thus, the presence of susceptible NHP and competent vectors in these places were considered to be the ideal scenario for the emergence of an epizootic, and control measures were readily instituted on these areas.

The conclusive diagnosis of the YF can be performed using different techniques such as viral isolation, molecular techniques (RT-PCR or RT-qPCR), and identification of the YFV antigen in the tissues (immunochemistry) [21]. As described by many studies, it was possible to observe some histopathological findings that are characteristic of a classic injury caused by the YFV, which is usually associated with the disease in humans (midzonal lytic necrosis, apoptotic bodies such as Councilman-Rocha Lima bodies, steatosis, and scarce paucicellular inflammation) [7, 21, 50–55].

Histopathological analysis by H&E staining in the liver cannot confirm the disease, as it may only suggest that an YFV infection is present. Immunohistochemistry can detect the presence of the virus in the tissues, and it is considered to be a confirmatory test for this virus. Before the advent of molecular techniques, this approach was considered to be the method of choice to diagnose YF [56, 57]. Thus, immunochemistry is considered to be part of the official surveillance and diagnosis for YF disease in humans and NHPs. Additionally, this technique assumes greater importance in a postmortem diagnosis, when much time has passed from death until sample collection, because these postmortem tissues are not suitable for use in molecular biology techniques. Immunohistochemistry is an important tool for the surveillance service, and it contributes to early detection of YFV in a region that is undergoing an outbreak [42].

Histopathological tests are important to make the correct diagnosis; however, the molecular techniques have improved and provided agility to the diagnosis and surveillance of the YFV. Molecular investigations performed in this study showed that four YFV RNA sequences, which were amplified from four different animals, were identical to each other and indicated that the same strain was circulating in the region during the study period. Similarly, all four RNA sequences were identical to the viruses detected in Espirito Santo state, KY885000 (strain ES-504/BRA/2017) and KY885001 (strain ES-505/BRA/2017), which were isolated in February 2017 [32]. This result showed that the same YFV was circulating in most of the southern part of Brazil.

According to surveillance studies by the Ministry of Health between 2014 and 2016, YFV has been spreading into the Amazon region and to other areas of the country in a southeastern direction, and it arrived in the central, western, and southeastern parts of the country between 2015 and 2016 [21, 38, 40]. In São Paulo state, YFV was first detected in the northwestern region and moved eastward, to the northeastern region of the state, where the study site was located. In this area, human YFV transmission was avoided because of the rapid implementation of the control measures described above, aiming to quickly detect the presence of the virus in the NHP population and consequently stop virus amplification. This was accomplished by the combined efforts of epidemiologists, veterinarians, field workers, and a solid infrastructure of virology laboratories and lab personnel. However, in the northern part of São Paulo state, especially in the area in which epizootics originated in the southern part of Minas Gerais state, prevention of YF in the human population was not as efficient, probably because it took too long to implement strategies such as the ones described above for this study site. The presence of islands of preserved forested areas, susceptible NHP, and an abundance of vectors resulted in a rapid southward dissemination of this virus in epizootic proportions, starting in Minas Gerais state and reaching the metropolitan region of São Paulo city in October 2017, when cases of NHP were detected in municipal parks [36]. Human cases detected in this region were associated with a low level of vaccine coverage. This type of epidemiological situation, where the YFV circulated within the Atlantic Forest biome and spread up to the coast of São Paulo state, had not been recorded since the 1930s [21, 39].

The recent YF outbreak that occurred in Brazil shows that a clear change in the pattern of the YFV sylvatic transmission cycle has recently emerged, showing a possible adaptation of this virus to areas that are in close proximity to urban environments where sylvatic cycle vectors and NHPs are increasingly found in association with humans. This allows for total conditions to perpetuate the YFV sylvatic cycle, as observed in the epizootic in 2016/2017. Additionally, environmental policies must be implemented in green protected areas and places with large NHP populations where the YFV circulated, and new public health policies must be formulated, such as new surveillance strategies (permanent active surveillance and monitoring of NHPs, vectors, and others animals in hot spot areas) because of the changing patterns that have been recently established for YFV. Ultimately, all these policies must take into consideration the possible reurbanization of YF in Brazil, and widespread vaccination should be advised. However, YF reurbanization has not happened, and all current YF human cases had a significant interaction with rural areas.

Although Brazilian health authorities have implemented surveillance actions for YF, our study shows the vulnerability of the available Health System to the occurrence of diseases that were previously considered to be controlled. Additionally, it underscores the requirement for integrated and multiprofessional actions between humans, animals, and environmental interfaces, and most importantly, the control actions must be rapidly implemented to avoid virus dissemination to disease-free areas.

## Figures and Tables

**Table 1:** Primers used in RNA detection by RT-qPCR and sequencing.

Name	Use	Sequence
YF.For	qPCR	GTGACAGCCTTGGCCATT
YF.Rev	qPCR	AGGCTGGGCCAACAGCCA
YF.FAM	qPCR	6FAM-GAAGCAACATGACGCAACGA-TAMRA
YF.Seq.For	Sequencing	ATCGTTCGTTGAGCGATTAGC
YF.Seq.Rev	Sequencing	CCAGTGCTGGGGCACTTGTCATT

**Table 2:** Nonhuman primate epizootics with collected samples and laboratory results, from November 2016 to April 2017.

Sequence number of notifications	Animal ID	Date of necropsy	Species	Results RT-qPCR
01	Monkey #1	04/11/16	*Alouatta caraya*	Positive
02	Monkey #2	04/11/16	*Alouatta caraya*	Positive^*∗*^
04	Monkey #3	30/11/16	*Alouatta caraya*	Negative
09	Monkey #4	07/12/16	*Alouatta caraya*	Positive
10	Monkey #5	11/12/16	*Alouatta caraya*	Positive
11	Monkey #7	08/01/17	*Sapajus nigritus*	Negative
13	Monkey #9	13/01/17	*Alouatta caraya*	Positive
14	Monkey #6	13/03/17	*Alouatta caraya*	Negative
15	Monkey #8	26/04/17	*Callithrix jacchus*	Negative

^*∗*^RT-qPCR performed by Adolfo Lutz Institute of São Paulo.

**Table 3:** Real-time RT-PCR results for yellow fever virus in several biological tissues of nonhuman primates of the *Alouatta caraya* species found dead in study sites between November 2016 and April 2017.

Tissue/organs	Monkey #1	Monkey #4	Monkey #5	Monkey #9
Blood	N/A	N	P	P
Serum	P	N/A	N/A	N/A
Brain	N/A	P	P	P
Heart	N/A	P	P	P
Lung	N/A	P	P	P
Liver	N/A	P	P	P
Stomach	N/A	N/A	N/A	P
Spleen	N/A	P	P	P
Large intestine	N/A	N/A	N/A	P
Mesenteric lymph node	N/A	N/A	N/A	P
Adrenal	N/A	N/A	P	P
Kidneys	N/A	P	P	P
Urinary vesicle	N/A	N/A	N/A	P
Gonads (testicle)	N/A	N/A	N	P

P, positive; N, negative; N/A, no available samples.

## Data Availability

The data used to support the ﬁndings of this study are included within the article.
